# Development and Validation of a Low-Cost Simulation Tool for Practical Training in Diagnostic Bronchoscopy for Otolaryngology Postgraduates

**DOI:** 10.7759/cureus.106843

**Published:** 2026-04-11

**Authors:** Yallanti Jothir Surya Teja, Lokesh Kumar Penubarthi, Dinesh Kumar V, Joel A Sherry, Anbarasi Madoure

**Affiliations:** 1 Otorhinolaryngology, Jawaharlal Institute of Postgraduate Medical Education and Research, Puducherry, IND; 2 Anatomy, Jawaharlal Institute of Postgraduate Medical Education and Research, Puducherry, IND

**Keywords:** bronchoscopy, clinical competence, medical education, medical residency, otorhinolaryngology, patient simulation

## Abstract

Objectives

The objectives of this study are to develop and validate a low-cost, low-fidelity simulation model for diagnostic bronchoscopy training among otolaryngology postgraduates (PGs) and to assess realism, educational utility, and learner confidence.

Methods

We constructed a simulation model replicating pediatric airway anatomy using locally available materials (INR 1,000). Twenty-six otolaryngology residents participated in structured simulation sessions. Face, content, curriculum, and transfer validity were evaluated using a five-point Likert scale survey. Statistical analysis employed the Friedman and Wilcoxon signed-rank tests.

Results

The participants reported high validity scores across all domains (median ≥ 4), with significant post-training confidence improvement (p < 0.001). No difference in scores existed between residents from different training years across validity domains (p > 0.05).

Conclusion

This low-cost bronchoscopy simulator effectively improves procedural confidence and aligns with curricular needs. It provides a practical and scalable training solution for resource-limited settings and early-stage residency programs.

## Introduction

A significant development in the field of medical education has been the incorporation of simulation models into academic curricula. Patient safety concerns, a crucial issue highlighted by the seminal Institute of Medicine report in 1999, have been the primary catalyst of this change. According to the report, between 44,000 and 98,000 deaths per year are attributed to medical errors, which impact roughly 3% of hospitalized patients in the USA. According to complementary results from the Harvard Medical Practice Study, adverse events happened to 3.7% of hospital inpatients, with 13.6% leading to death and 27.6% attributable to negligence [1]. These figures demonstrate the critical need for clinical competency/practical training and theoretical knowledge to be given equal weight in medical education.

A key strategy in addressing this need is simulation-based training (SBT), which provides an adequate environment for learners to gain procedural competence and confidence. It lowers healthcare expenses, promotes skill development, and improves patient safety without endangering the patient [1]. In otolaryngology, SBT is especially important where even simple procedures such as emergency tracheostomy, upper aerodigestive tract foreign body removal, and abscess drainage can save human lives. Performing such interventions in real-life settings can be stressful and intimidating for residents and novices. Simulation models ensure that trainees are well-prepared, reduce stress, and boost confidence. For example, it has been demonstrated that using peritonsillar abscess drainage training models greatly increases residents' procedural confidence in otolaryngology [2].

A common otolaryngology procedure, critical in pediatric populations, is diagnostic bronchoscopy. Airway anatomy can be directly visualized, airway dynamics can be evaluated, and diagnostic and therapeutic procedures such as foreign body removal, biopsies, lavage fluid collection, medication administration, and help with challenging intubations may be carried out by practicing on a simulation model [3-5]. A thorough understanding of airway anatomy is necessary for a safe and successful bronchoscopy, underscoring the significance of organized simulation training [6].

However, the high cost of existing high-fidelity bronchoscopy simulators, which frequently exceed US$2590 (roughly INR 220,000), restricts their use, especially in settings with limited resources and institutions with sizable student bodies. Consequently, it becomes tough to provide sufficient opportunities for practical training. Low-fidelity simulation models are a cost-effective substitute for teaching intricate procedural skills, according to recent research [7,8]. These models have several benefits, including being learner-centered, lowering stress, standardizing task performance, and enabling impartial evaluation and feedback. Crucially, research has demonstrated that for airway management training, low-fidelity simulators can be just as successful as high-fidelity models [9-12]. They are particularly useful for widespread use due to their lower cost, simplicity of maintenance, portability, and ease of reproduction. The study aims to develop a low-fidelity bronchoscopy model and validate its effectiveness in training otolaryngology residents. The suggested model costs about INR 1,000, roughly 1% of the cost of high-fidelity alternatives. The study will assess the model's face validity, the degree to which users perceive realism; content validity, the applicability and relevance of teaching fundamental bronchoscopy skills; curriculum validity, how well it integrates into and aligns with current educational programs; and transfer validity, the extent to which well-acquired skills translate to clinical practice.

By evaluating these dimensions, the study aims to demonstrate the low-cost model as a practical and efficient tool for procedural training in otolaryngology.

## Materials and methods

Development of the model

We enumerated the required anatomical structures and reviewed standard textbooks and online sources on the anatomy of the trachea and bronchial tree [13-15]. The dimensions and angulations of the model are approximately those of a three-year-old child. We chose this age as children below three years were the most likely to undergo bronchoscopy in our institute. To give our residents an efficient training aid, the goal was to develop a model that faithfully resembles human anatomy, especially the trachea and bronchi.

**Figure 1:**
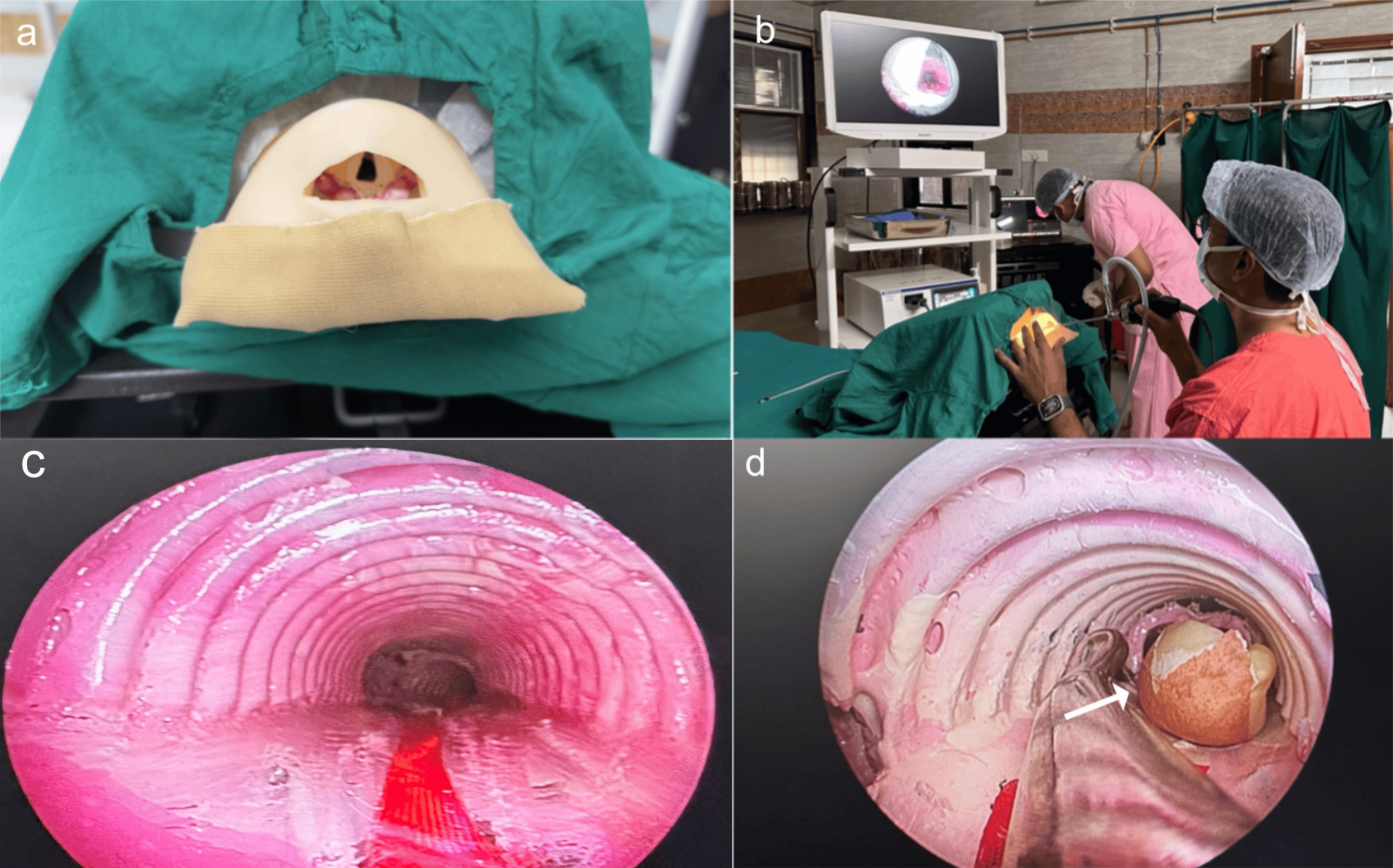
Gross and endoscopic view of the model (a) Gross appearance of the bronchoscopy model. (b) The training session for postgraduate residents in the skills laboratory. (c) The endoscopic view of the tracheal rings. (d) A peanut kept (arrow) and retrieved from the right bronchus using a grasper during the training session

We used a longitudinally sectioned corrugated drain pipe to resemble the endoscopic view of tracheal rings during bronchoscopy (Figure 1).

A 2 mL disposable syringe was heat-molded and shaped to resemble the primary bronchi. The intravenous flow set was used distally to resemble the dimensions of the secondary bronchi. The inner surface of the trachea and bronchi was lined with light-pink acrylic paint to make the endoscopic view more realistic. Meticulous care was taken to maintain exact anatomical angulations at the carina to give a more realistic experience while entering the right and left bronchi with an endoscope. We used protractors to measure the angulations between the trachea and bronchi precisely. At various levels, high-quality plumbing adhesives were used at the junctions between the trachea and bronchi. The trachea-bronchial tree was secured in a 25 × 32 cm plastic box filled with a polystyrene block cut precisely to fit the trachea-bronchial tree. Moldable iron strings secured to the polystyrene base within the box allowed us to maintain the proper angles. This box functioned as the model's foundation and allowed the observation of the endoscopic glow inside the lumen of internal structures while training. The dummy head was precisely positioned around the trachea-bronchial tree and fixed into a hole carved on one side of the box. We have added a layer of authenticity to the training experience by covering the transparent plastic box with a surgical gown to replicate the atmosphere of an operating room. The whole model was transferred to the skills laboratory to make it available to the postgraduates (PGs) for training and validation.

Inclusion and exclusion criteria

All otolaryngology postgraduate residents undergoing training in our department during the study period were considered eligible for participation. Postgraduate residents from any year of training (PG-1, PG-2, or PG-3) and senior residents who had recently completed postgraduate training in otolaryngology, who are willing to participate in the study and practice on the model session in person, were selected for the study. Residents were included irrespective of prior exposure to bronchoscopy, as the objective was to evaluate the educational utility of the model across different levels of baseline experience. We excluded residents who were not willing to provide consent to participate, those who were unavailable for the training session during the study periods, those who did not complete either the pre-test or post-test questionnaire, and those with incomplete or duplicate survey responses.

A total of 26 eligible participants satisfied the inclusion criteria and were analyzed.

Survey methodology

The survey was done in Jawaharlal Institute of Postgraduate Medical Education and Research (JIPMER), Puducherry, from June 2024 to October 2024. The Institutional Ethics Committee for Observational Studies of JIPMER issued approval JIP/IEC-OS/2024/306. A pre-test and post-test survey was employed to evaluate the training model's impact on residents' confidence. All otolaryngology residents who met the inclusion criteria and consented to participate were selected for the survey. Before the simulation session, each participant completed a pre-test survey assessing their self-reported confidence levels in performing bronchoscopy. This was administered via a structured Google Form (Google, Inc., Mountain View, CA), using a five-point Likert scale (1 = not confident at all; 5 = extremely confident) (Appendices). The survey captured prior exposure to bronchoscopy procedures, if any. A faculty member from the Department of Otorhinolaryngology briefly introduced the model and the procedure as shown in Figure 1.

Each participant was then given a 10-minute individual session to explore and practice with the model, ensuring adequate time to familiarize themselves with the anatomy and simulate endoscopic navigation. We provided an anonymous Google Form link to the participants to provide their score for each domain of the survey (face, content, and curriculum validity). Post training/practice, the participants were also asked to complete a post-test survey using the same Google Form link. Training sessions were distributed over five days to accommodate residents' schedules and minimize fatigue. All feedback was collected anonymously and compiled into an Excel (Microsoft Corp., Redmond, WA) spreadsheet for statistical analysis to assess the change in confidence and the overall efficacy of the simulation tool.

Statistical analysis

Data obtained from the pre-test and post-test questionnaires were entered into Microsoft Excel and analyzed using IBM SPSS version 29.0 (IBM Corp., Armonk, NY). Continuous variables such as age were planned to be summarized as mean ± standard deviation (SD) for normally distributed data or median with interquartile range (IQR) for non-normally distributed data. Categorical variables, including year of residency and prior bronchoscopy exposure, were summarized as frequencies and percentages. Responses to Likert scale items were treated as ordinal data and reported as median (IQR).

The distribution of survey responses was assessed using the Shapiro-Wilk test. As the data did not follow a normal distribution (p < 0.05), nonparametric tests were used for inferential analysis. The comparison of scores across the survey domains was performed using the Friedman test. When the Friedman test showed statistical significance, pairwise post hoc comparisons were carried out using the Wilcoxon signed-rank test with Bonferroni correction to adjust for multiple comparisons. The comparison of pre-test and post-test transfer validity (confidence) scores was also performed using the Wilcoxon signed-rank test. Differences in domain scores between the residents of different years of training were analyzed using the Kruskal-Wallis test. A two-tailed p value of less than 0.05 was considered statistically significant, except for Bonferroni-adjusted pairwise comparisons, where p < 0.008 was considered significant.

## Results

All 26 otolaryngology residents who underwent training on this model participated in validating the model's effectiveness. The demographic details of the participants are depicted in Table 1.

**Table 1:** Demographic distribution of the participants PG: postgraduate

Variable	Category	n (%)/Summary
Total participants	-	26
Age (years)	-	Median: 25 (range: 22-27)
Sex	Male	8 (30.8)
	Female	18 (69.2)
Level of training	Senior residents/post-PG	4 (15.4)
	PG-1	9 (34.6)
	PG-2	7 (26.9)
	PG-3	6 (23.1)
Prior bronchoscopy exposure	Yes	4 (15.4)
	No	22 (84.6)

**Figure 2:**
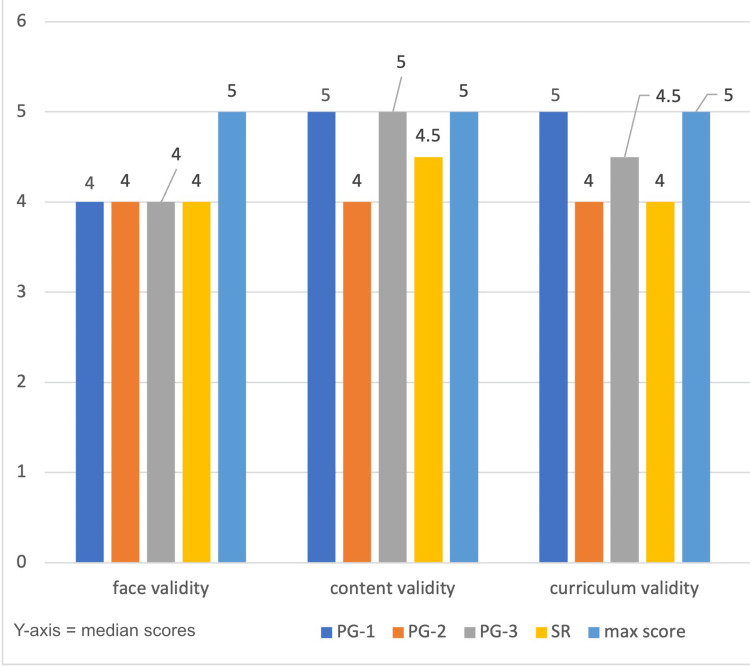
Median scores across all the domains The median scores' comparison between different training years for each of the three validation domains. The y-axis represents the median scores. The blue bar represents the maximum possible score (5) in each domain. Across all training levels, the scores were consistently high, with minimal variation, indicating broad agreement on the effectiveness and perceived value of the simulation model, regardless of the level of experience PG, postgraduate; SR, senior resident

The survey's four domains, face validity, content validity, curriculum validity, and confidence attained (transfer validity) following model simulation exercises, were answered by all participants. After the simulation exercise, all 26 participants completed a 17-question survey to assess the bronchoscopy model's face, content, curriculum, and transfer validity (confidence attained by the participant). The year-wise scores obtained by PGs to the maximum score in each domain are illustrated in Figure 2.

The median (IQR) scores given for each question in the survey by residents are illustrated in Table 2.

**Table 2:** Survey of each question in the different domains with a five-point Likert scale response Self-developed and validated questionnaire for the bronchoscopy model IQR: interquartile range

Statements	Strongly Disagree	Disagree	Neutral	Agree	Strongly Agree	Median (IQR)
Face validity						
1. The appearance of the endoscopic model is close to reality	0 (0)	0 (0)	15.3 (4)	61 (16)	23 (6)	4 (0)
2. Endoscopic trachea and bronchi look close to reality	0 (0)	0 (0)	0 (0)	69.2 (18)	30.7 (8)	4 (1)
3. Depth perception is realistic	0 (0)	3.8 (1)	3.8 (1)	57.6 (15)	19.2 (9)	4 (1)
4. Instrument application is realistic	0 (0)	7.6 (2)	27 (7)	30.7 (8)	34.6 (9)	4 (2)
5. The angulations at the carina are close to reality	0 (0)	0 (0)	11.5 (3)	53.8 (14)	19.2 (9)	4 (1)
Content validity						
1. This model is useful for teaching the endoscopic anatomy of the trachea and bronchi	0 (0)	0 (0)	11.5 (3)	38.4 (10)	50.0 (13)	4.5 (1)
2. This model is useful for teaching diagnostic bronchoscopy	0 (0)	0 (0)	7.6 (2)	34.6 (9)	57.6 (15)	5 (1)
3. This model is useful for improving hand-eye coordination	0 (0)	3.8 (1)	11.5 (3)	34.6 (9)	50.0 (13)	4.5 (1)
4. This model is useful for teaching bronchial foreign body removal	0 (0)	3.8 (1)	7.6 (2)	42.3 (11)	46.1 (12)	4 (1)
5. This model is useful as an overall training tool	0 (0)	0 (0)	3.8 (1)	50.00 (13)	46.1 (12)	4 (1)
Curriculum validity						
1. Skills learned are transferable to the bronchoscopy room	0 (0)	7.6 (2)	11.5 (3)	57.6 (15)	23 (6)	4 (0)
2. This model can be incorporated into the training curriculum	0 (0)	0 (0)	0 (0)	53.8 (14)	46.1 (12)	4 (1)
3. I would recommend this model to other trainees	0 (0)	0 (0)	3.8 (1)	30.7 (8)	65.38 (17)	5 (1)

We compared the median scores residents gave across the four survey domains using the Friedman test, which showed a statistically significant difference between the domains. Post hoc analysis using the Wilcoxon signed-rank test with Bonferroni correction revealed that the scores for face validity, content validity, and curricular validity were all significantly higher than the pre-test scores of transfer validity (which reflects the confidence gained by the participants), with p < 0.008.

**Figure 3:**
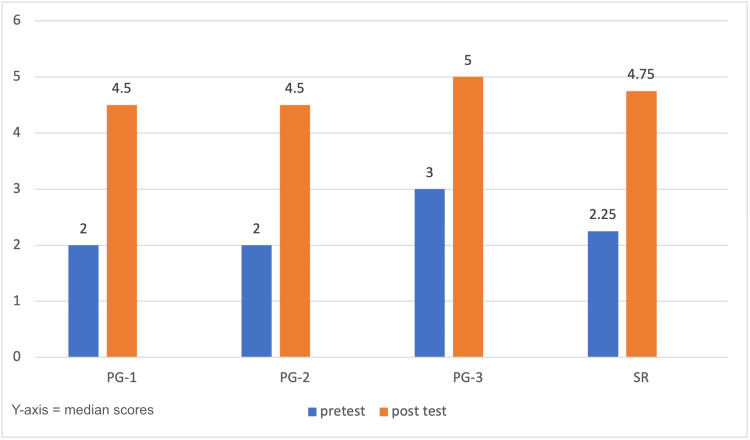
Graph depicting the transfer validity The median pre- and post-test scores of transfer validity are illustrated. The y-axis represents the median scores obtained. There was a significant difference in the scores, emphasizing the importance of training on the simulation model before doing the actual bronchoscopy procedures PG, postgraduate; SR, senior resident

The pre-test scores for transfer validity (confidence levels) (Table 3) were compared to the post-test transfer validity scores for all the participants. There was a significant difference between the scores across all postgraduates of different clinical years (p < 0.001; Wilcoxon signed-rank test) (Figure 3).

**Table 3:** Transfer validity pre-test scores IQR: interquartile range

Statements	Not at All Confident	Slightly Confident	Moderately Confident	Very Confident	Extremely Confident	Median (IQR)
1. How confident are you in handling a bronchoscopy case?	15.38 (4)	53.8 (14)	27 (7)	3.8 (1)	0 (0)	2 (1)
2. How confident are you in performing each step of the bronchoscopy?	23 (6)	38.46 (10)	30.7 (0)	7.6 (2)	0 (0)	2 (1)
3. How confident are you in transferring skills to a real situation?	19.23 (5)	27 (7)	23 (6)	20.7 (8)	0 (0)	3 (2)
4. How confident are you in removing foreign bodies from the trachea and bronchi?	23 (6)	46.1 (12)	19.23 (5)	11.5 (3)	0 (0)	2 (1)

This highlights the importance of the simulation model and practical training to build confidence in the postgraduates before they face actual day-to-day emergency bronchoscopy procedures.

## Discussion

The findings of our study emphasize the critical role of low-cost, easily reproducible, low-fidelity simulation tools in otolaryngology training. Our bronchoscopy model was developed using readily available materials at just INR 1000 (approximately US$12). In contrast, commercially available bronchoscopy simulators such as the Nakhosteen Bronchoscopy Model (Broncho Boy) by Coburger Lehrmittelanstalt (CLA) (Coburg, Germany) and the Bronchoscopy Model by Koken Co., Ltd. (Tokyo, Japan) are priced between US$3000 and US$5000, making them prohibitively expensive for widespread use, particularly in resource-limited settings [16,17]. The cost barrier limits access to simulation-based training in many healthcare sectors. Especially in developing countries, there is an urgent need to design affordable and scalable simulation models that can be adopted across medical schools and healthcare institutions to ensure equitable access to procedural training [18].

The integration of simulation into postgraduate educational programs is becoming increasingly vital. There is a heightened need for validated, reproducible simulators to train budding surgeons in commonly performed endoscopic procedures and in managing rare and high-risk complications, as recommended by the Accreditation Council for Graduate Medical Education (ACGME)/Residency Review Committee (RRC) guidelines [19]. Rising medical training costs have reduced residents' exposure to critical procedures. Life-saving interventions, such as removing foreign bodies from the airway, remain a crucial component of the otolaryngology curriculum. However, allowing novice residents to perform such procedures directly on patients in the operating room is highly risky and potentially harmful [20]. It is also crucial to differentiate airway foreign bodies from seasonal pneumonias, especially in children, as the latter can masquerade as chronically retained foreign bodies in the airway.

A systematic review by Pankhania et al. identified 18 low-cost models in otology, head and neck surgery, laryngeal surgery, rhinology, and tonsillectomy [21]. However, only 22.2% of these models attempted to formally demonstrate educational impact, and validation was rarely performed. These findings underscore the urgent need to standardize validation methods and critically evaluate the educational value of low-cost simulators, reinforcing the significance of research efforts such as ours.

We have validated our model among postgraduates and senior residents with varying years of experience in four domains. We found that postgraduates and senior residents were satisfied and gave good scores on the appearance (face), content, and adoption of the model into the ENT curriculum (median scores of more than 4 out of 5 for all domains). Simulation allows students to build and hone their procedural skills through repeated haptic and constructive feedback. Simulation models enable new residents and undergraduate students to rehearse clinical procedures, thus encouraging competency development early in their careers [22,23].

Similarly, Santa Maria et al. proved that commercially purchased bronchoscopy simulators adequately measure bronchoscopy competence at different levels of postgraduate training [24]. Their prospective cohort study with 32 otolaryngology residents and four faculty instructors showed high correlations between training year and efficiency at diagnostic bronchoscopy, foreign body extraction, and tracheal lesion biopsy tasks. Additionally, residents highly rated simulator realism with a mean rating of 4.1 out of 5. These results corroborate the idea that well-designed simulation at low expense can advance critical skill development in a secure and controlled setting. In this study, we thoroughly evaluated the validity of a low-cost model for a bronchoscopy simulator across different domains such as face validity, content validity, and curriculum validity.

Face validity refers to the extent to which a simulation model accurately replicates real-life anatomical structures and procedural settings. In our study, this was ensured by meticulously replicating anatomical dimensions and landmark points, which were reviewed and validated by subject experts from the Departments of Anatomy, Pulmonary Medicine, and Otorhinolaryngology. All 26 residents unanimously endorsed the model's realism, particularly appreciating features such as the endoscopic view of the tracheobronchial tree, depth perception, carinal angulations, and the ease of instrument handling. The median (IQR) face validity score was 4 (zero), indicating strong agreement across participants, irrespective of their level of training. Special attention was given to select materials that closely mimic human anatomy. The epiglottis was crafted using moldable clay, while the vocal cords were fabricated from Silastic sheets to enhance visual realism during bronchoscopy. Tracheal rings were represented using longitudinally sectioned corrugated polyvinyl chloride (PVC) drain pipes of suitable dimensions. Additionally, the inner surfaces of the trachea and main bronchi were hand-painted with acrylic colors to replicate the natural appearance of the human tracheobronchial mucosa.

Content validity refers to the degree to which a simulation model accurately reflects the knowledge and skills required in the real clinical setting, particularly regarding cognitive and psychomotor domains such as anatomical orientation, hand-eye coordination, and instrument handling. Our study assessed content validity as a core domain assessed through structured feedback from postgraduate residents. Encouragingly, this domain received the highest overall score among all evaluated aspects, with a median score of 4.5, reflecting strong consensus on its educational utility.

The primary objective of our model was to function as a teaching tool, especially for novice learners, by replicating endoscopic anatomy and procedural nuances relevant to foreign body removal. The simulation incorporated a peanut lodged in the tracheobronchial tree, mimicking a common real-life airway obstruction scenario encountered in pediatric otolaryngology. This simulation model facilitated a realistic and engaging training experience that promoted spatial orientation and safe instrument navigation.

Notably, first-year residents rated the model more beneficial than their senior counterparts (median scores of 5 versus 4.5, respectively), likely due to their limited clinical exposure. This is consistent with the literature, where simulation-based training has significantly enhanced procedural preparedness and confidence among early trainees [25]. Simulators are essential educational tools to build foundational skills before transitioning to live patient procedures, reducing patient risk and improving educational outcomes [26].

Furthermore, bronchoscopy simulators have demonstrated effectiveness in teaching basic airway anatomy and complex procedural steps, with several studies confirming their role in improving technical competence and procedural safety in novice learners [27]. The positive perception of our model's content validity supports its integration into structured training programs, particularly for junior residents needing early exposure to critical airway management skills.

Curriculum validity assesses how well a simulation model aligns with the intended learning objectives and practical skills outlined in current medical education curricula. In our study, the participants generally acknowledged the value of the model in supporting key competencies relevant to airway management and foreign body removal, which are fundamental in otolaryngology training. However, some participants felt that certain aspects of the simulation did not fully translate into real-world clinical settings, particularly the neck flexibility, artificial secretions inside the endobronchial lumen, etc. These factors may hinder the realistic maneuvering of the bronchoscope, especially when replicating subtle head positioning and dynamic airway alignment required during live procedures. These design limitations underscore the importance of continuous iterative development to enhance model realism while preserving cost-effectiveness, a challenge echoed in previous simulation studies.

Despite these limitations, participant feedback affirmed the model's overall educational value. Most residents "strongly recommended" the model for peer training (median {IQR} score = 4 {0.5}), reflecting a high level of user satisfaction and endorsement. This widespread acceptance supports its potential integration into early residency training programs, particularly as a preparatory tool before clinical exposure. Future development will address the mechanical shortcomings by exploring more flexible, lifelike materials, such as silicone composites and 3D-printed biomimetic structures, without significantly increasing cost. Such improvements could enhance face and curriculum validity, expanding the model's relevance and applicability within clinical training programs.

Transfer validity, also called confidence validity, evaluates how training with a simulation model translates into increased self-confidence among learners in performing the procedure in real-life clinical scenarios. In our study, the participants exhibited notably low confidence levels in the pre-test phase, regardless of their year of residency. This reflects the inherent anxiety and unfamiliarity associated with high-risk procedures, such as rigid bronchoscopy, when not adequately rehearsed. Following the simulation-based training session using our bronchoscopy model, post-test scores showed a statistically significant improvement compared to pre-test scores (p < 0.001; Wilcoxon signed-rank test) (Figure 3). This clear elevation in self-reported confidence levels underscores the educational efficacy of simulation-based training. Similar results were shown when a low-cost simulator for nasal endoscopy was developed and validated, reiterating the need to further develop low-fidelity models and their adoption in the curriculum [28].

Simulation training has consistently enhanced procedural skills and improved learner confidence, especially in high-stakes and infrequently encountered scenarios. Pre- and post-training assessments serve as objective tools to measure this impact. A systematic review by Issenberg et al. highlighted that effective simulation is closely associated with feedback and repetitive practice, both reinforced through structured pre- and post-test evaluations [26]. Cook et al. similarly emphasized that learners who undergo simulation training demonstrate superior skill acquisition, knowledge retention, and confidence compared to those trained via traditional methods [29]. The improvement observed in our study validates the critical role of simulation in bridging the gap between theoretical knowledge and clinical competence, especially for emergency airway procedures. It further advocates for integrating such low-cost yet effective models in structured otolaryngology training curricula.

Limitations

Firstly, validation was subjective and based on Likert scale answers. The low-cost model must be pitted against existing commercially available ones to have a more objective assessment. Secondly, learners were not permitted to practice until they reached a certain level of proficiency; thus, a proficiency gain curve could not be measured. Finally, the rigidity of the model restricted natural hand movements. Some of the participants also proposed additions, such as enhanced flexibility, the inclusion of nasal and oral cavities for trans-nasal simulation, and the use of LED lights for increased visualization. Although these additions would enhance realism, they might undermine the low-cost aspect of the model. Some other proposals, such as increasing the flexibility of the epiglottis or simulating more difficult foreign body removals, would involve more technical sophistication and a greater cost.

## Conclusions

In general, the participants reacted favorably to the low-cost model of bronchoscopy training, awarding high marks for face, content, and curriculum validity. Most felt that the model closely simulates the endoscopic anatomy of the trachea and bronchi and is a good training tool. Its advantages are simplicity, anatomical accuracy, ease of reproduction, and cost-effectiveness. Though high-end capabilities were suggested, the current model adequately covers the primary training needs of early-stage ENT residents. Enhancement in the future must be balanced against technical, cost, and feasibility factors. However, this low-cost simulator is a valuable addition to the training program, with a practical and realistic platform for skill acquisition.

## Appendices

The link for the Google Form used for the survey of the model is as follows: https://docs.google.com/forms/d/e/1FAIpQLSchibgqKio0OxI8n4MiH5jUSWlktH5O7piujAmHrImyYbVzyQ/viewform?usp=sharing. Table 4 shows the survey used for validation.

**Table 4:** Survey created for validation

Question	Responses				
	Likert score				
Face validity	1	2	3	4	5
The appearance of the endoscopic model is close to reality	Strongly disagree	Disagree	Neutral	Agree	Strongly agree
Endoscopic trachea and bronchi look close to reality	Strongly disagree	Disagree	Neutral	Agree	Strongly agree
Depth perception is realistic	Strongly disagree	Disagree	Neutral	Agree	Strongly agree
Instrument application is realistic	Strongly disagree	Disagree	Neutral	Agree	Strongly agree
The angulations at the carina are close to reality	Strongly disagree	Disagree	Neutral	Agree	Strongly agree
Content validity					
This model is useful for teaching the endoscopic anatomy of the trachea and bronchi	Strongly disagree	Disagree	Neutral	Agree	Strongly agree
This model is useful for teaching diagnostic bronchoscopy	Strongly disagree	Disagree	Neutral	Agree	Strongly agree
This model is useful for improving hand-eye coordination	Strongly disagree	Disagree	Neutral	Agree	Strongly agree
This model is useful for teaching bronchial foreign body removal	Strongly disagree	Disagree	Neutral	Agree	Strongly agree
This model is useful as an overall training tool	Strongly disagree	Disagree	Neutral	Agree	Strongly agree
Curriculum validity					
Skills learned are transferable to the bronchoscopy room	Strongly disagree	Disagree	Neutral	Agree	Strongly agree
This model can be incorporated into the training curriculum	Strongly disagree	Disagree	Neutral	Agree	Strongly agree
I would recommend this model to other trainees	Strongly disagree	Disagree	Neutral	Agree	Strongly agree
Transfer validity					
How confident are you in handling a bronchoscopy case?	Not at all confident	Slightly confident	Moderately confident	Very confident	Extremely confident
How confident are you in performing each step of the bronchoscopy?	Not at all confident	Slightly confident	Moderately confident	Very confident	Extremely confident
How confident are you in transferring skills to a real situation?	Not at all confident	Slightly confident	Moderately confident	Very confident	Extremely confident
How confident are you in removing foreign bodies from the trachea and bronchi?	Not at all confident	Slightly confident	Moderately confident	Very confident	Extremely confident
